# N-of-1 Design and Its Applications to Personalized Treatment Studies

**DOI:** 10.1007/s12561-016-9165-9

**Published:** 2016-09-06

**Authors:** Tailiang Xie, Zhuoxin Yu

**Affiliations:** Brightech International, 285 Davidson Avenue, Suite 504, Somerset, NJ 08873 USA

**Keywords:** N-of-1 design, Personalized medicine, Traditional Chinese medicine, Cross-over, Simulation

## Abstract

N-of-1 trial is a type of clinical trial which has been applied in chronic recurrent conditions that require long-term non-curative treatment. In this type of trials, each patient will be randomly assigned to one of the treatment sequences and repeatedly crossed over two or more treatments of interests. Through this cross-comparing method (cross-over phase), investigator can identify an optimal treatment (medicine or therapy) for the patient and treat the patient with the optimal treatment in an extension phase. This design could efficiently reduce the placebo effect, which is often seen in clinical trials, and maximize the true treatment effect. This type of design has been used in some traditional Chinese medicine (TCM) clinical trials lately. However, it brings some challenges for collecting and analyzing the data. Research on statistical methodology of this type of design is rarely found in the literature. The goal of this research is to discuss the application of the N-of-1 design to personalized treatment studies. We will demonstrate a real study conducted in TCM and present some theoretical and simulation results.

**Electronic supplementary material:**

The online version of this article (doi:10.1007/s12561-016-9165-9) contains supplementary material, which is available to authorized users.

## Introduction

In controlled clinical trials, patients were enrolled according to the inclusion and exclusion criteria. The purpose for setting these criteria is to ensure a homogeneous patient population to be studied. In study design stage, we define inclusion and exclusion criteria according to the knowledge of the medical conditions and prognostic factors of the disease to be treated. However, we often find out that an active treatment in a well-controlled study only works for a subset of patients, although the therapeutic mechanism is well characterized. For example, molecule-targeted cancer drugs are only effective for certain percentage of patients even though they had the tumor-expressing targets. Some newly developed drugs may be abandoned because no significant improvements have been detected across a population, whereas it is highly possible that subgroups of patients could benefit from them [1]. If drugs A and B are both proven to be effective to a disease, however, we often see that some patients obtained treatment benefit from drug A, some obtained treatment benefit from drug B and some did not have any benefit from either of them. Even for a highly effective drug, the level of treatment benefit for different people may vary. There may be many reasons behind it. One of them may be the genetic differences in patients or some unknown prognostic factors of the disease [2, 3]. For patients who did not respond to the drug, there might be some characteristics or genetic information that may interfere with the drug effect which had not been known yet at the time of the study. These phenomena suggest us reconsider the medical research and drug development and pay more attention to personalized medicine or treatment.

The goal of personalized medicine is to achieve the optimal clinical outcome by steering patients to the right drug at the right dose and right timing [1]. In recent years, there are extensive researches which studied personalized medicine/treatment, and most of the approaches focus on patients’ known prognostic factors, genetic information, or biomarkers. The review paper by Zhao et al. nicely summarized recent researches and statistical methods for personalized medicine/treatment. There are two types of approaches for studying personalized medicines, the single-stage study and multi-stage study. In single-stage study, one identifies a personalized treatment based on patients’ baseline information to maximize the treatment benefit or minimize the risk due to the treatment, whereas in the multi-stage study, one identifies a personalized treatment based on a sequence of treatment approaches at each stage so that treatments are adaptive to patients’ characteristics, disease histories, and other evolving biomarkers [1]. For multi-stage study, Murphy and others have published a number of papers on statistical methodology of adaptive treatment strategies, also called dynamic treatment regimes, which are sequential treatment assignments for individual patients [4–8]. In this manuscript, we will introduce a new statistical method for personalized medicine, the N-of-1 design, which could be considered as a special kind of multi-stage adaptive strategy. In the N-of-1 design, two or more treatments are provided to a patient in cross-over fashion and the best one to the patient will be identified and picked for long-term follow-up treatment.

N-of-1 design is composed of a series of pairs of treatments [9]: within each pair, there are always a period on experimental treatment (A) and another period on alternative treatment or placebo (B). The order of treatments A and B is randomly determined. By evaluating the differences between treatments A and B, the main purpose of N-of-1 design is to find the best treatment or to determine whether a certain treatment is truly effective for a particular patient. N-of-1 design was first systematically explained in psychology [10] and may be rarely used in clinical trials so far. But N-of-1 design drew some attention in the medical field since 1986 [11] and since then have been applicable for some chronic diseases with symptomatic conditions, such as arthritis [12–15], asthma [16, 17], fibromyalgia [18, 19], insomnia [20], and attention deficit hyperactivity disorder [21–23]. N-of-1 design could also be used to evaluate the safety of study drug to improve patient adherence to clinical trial [24]; to improve patient management and save costs for chronic diseases [25]; to examine surgical procedures [26]; and to help decision making for treatments that might have adverse consequences or costs [27].

Earlier N-of-1 trials always focused on very limited number of patients (usually one single patient) instead of a large population of patients [28–30], so N-of-1 design falls well in the scope of personalized medicine/treatment. In recent years, in some publications, individual N-of-1 trials were aggregated to evaluate population treatment effects and provide the robustness of a regular RCT trial, since each patient contributes more than one set of perfectly matched data. In a recent review [31], it was reported that out of 108 included n-of-1 trials over 25 years (including single-patient trials and multiple-patient trials), about half of them used *t* test and the other half used only graphical comparison (plotting responses over time and determining efficacy by visual inspection) with no statistical analysis; of the 60 multiple-patient trials, 43 % reported on a pooled analysis, 23 % of which used Bayesian methodology and the others used frequentist statistics. A few studies [27] used hierarchical Bayesian method [32] which could combine the results from single-patient trials and get posterior estimates of the population treatment effects as well as the individual patient treatment effects, using either normal likelihood distributions [33] or binomial likelihood distributions [34]. The conclusions drawn may differ between various Bayesian analyses due to sensitivity to the informative prior distribution used [34].

**Fig. 1:**
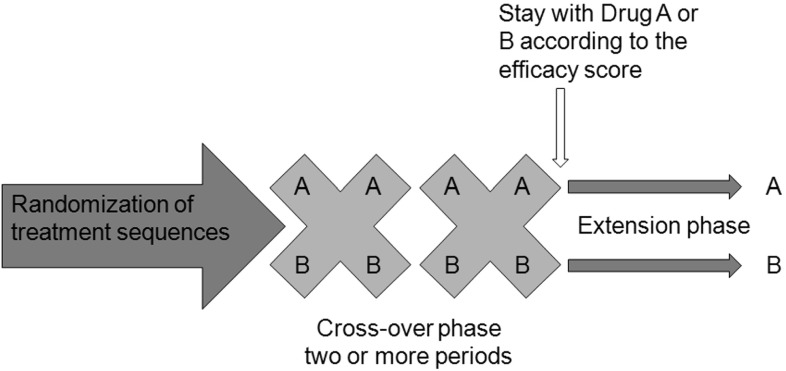
N-of-1 design without placebo

Here we propose an alternative method to analyze the data and compare the treatment effect. We also believe that N-of-1 trial could not only be carried out on one patient at a time, but it also has great potential to be incorporated to traditional randomized trials, which could be beneficial to a larger population of patients. Thus we developed a brand new clinical trial design which consists of two phases: a cross-over phase and an extension phase (Fig. 1). The cross-over phase actually follows N-of-1 design, and in this phase, patients take two potentially active treatments A and B alternately. At the end of cross-over phase, treatments A and B are evaluated by treatment effects (always by scores) for each patient. For one particular patient, if treatment A is better than B, this patient stays in treatment group A; and the same rule applies to patients in favor of treatment B. Then all the patients with successful assignments of treatment A or B will enter extension phase, in which a patient will only be given one treatment, A or B, according to the assignment. At the end of the trial, information gathered in both cross-over phase and extension phase will be used to analyze the treatment effects.

From statistical point of view, there are quite a few questions for this new design. For example, how to estimate the sample size to ensure the powers covering both phases? How to define the overall endpoint and how to analyze it? How many cross-overs are needed to avoid a false positive decision? In this manuscript, we try to address these questions.

## Method

### A Motivational Example and Concept

**Fig. 2:**
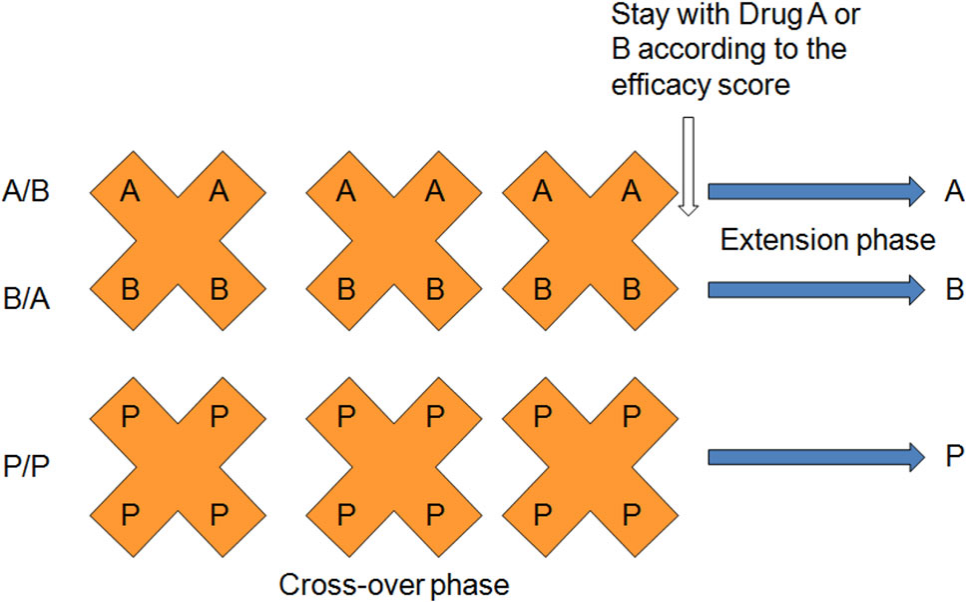
Generalization of the N-of-1 design with placebo

Assume that we are studying two active drugs (Drugs A and B) on a disease population and a 10 % placebo (P) response was observed for this population. The entire study population contains two subgroups U ($r^*100\%$ of people) and $\text {V}((1-r)^*100\%$ of people). These two subgroups react differently on Drugs A and B. Let us assume that Drug A works on subgroup U with effect of 0.5 (full effect), but it works on subgroup V with effect of only 0.1 (the same as placebo). On the contrary, Drug B works on subgroup U with effect of only 0.1, but it works on subgroup *V* with full effect of 0.5. If $r=0.5$, the effects of drugs A and B on the whole population are both 0.3 and $\Delta _\mathrm{AP} = \Delta _\mathrm{BP} = 0.2$, where $\Delta _\mathrm{AP}$ and $\Delta _\mathrm{BP}$ denote the treatment differences of A or B versus placebo, respectively. Using the N-of-1 design, in the ideal case, if we could correctly apply Drug A to subgroup U and Drug B to subgroup V, we have $\Delta _\mathrm{AP} = \Delta _\mathrm{BP} = 0.4$, which enhances the treatment benefit greatly by correct assignment of the drugs. This leads to a generalization of the N-of-1 design to active drug/placebo-controlled study as illustrated in the following diagram (Fig. 2).

In this design, patients will be randomly assigned to treatment sequences A/B, B/A, and P/P at 1:1:1 ratio. Treatments will be crossed over according to the assigned sequence. The cross-over can be repeated for *k* ($k \ge 1$) times (say 2 or 3 times) so that the treatment efficacy (e.g., symptom relief score) can be sufficiently identified. At the end of the cross-over phase, patient’s efficacy score will be reviewed and patient will be given the treatment with better score in the extension phase. Of course, the criteria of “better” could be predefined clinically (For example, a 10 % better is considered clinically meaningful). Patients assigned to P/P will be handled in the same way. There will be no impact to the treatment assignment in the extension phase for the P/P patients. The entire process should be handled in blinded fashion.

Whether to include a P/P depends on the objectives of the study. If A is an experimental drug, B is an approved drug and both are for the same indication, the goal is to assess non-inferiority of A versus B, then including P/P will enable us to demonstrate the drug sensitivity (or “assay” sensitivity) in the sense that Drug A must be superior to placebo. If both A and B are approved drugs, the goal is to evaluate whether A is better than B or vice versa or they are equivalent or this is a personalized treatment study, then it is not necessary to include the P/P sequence.

We will discuss the pros and cons and statistical challenges of these scenarios in the following sections.

### Sample Size Consideration

Since the design consists of two parts (cross-over and extension), we need to estimate the sample size so that the sufficient statistical power for tests in cross-over and extension phases would be warranted. In other words, we need to calculate the sample size required for cross-over phase and the sample size for extension phase as well.

For the cross-over phase, the sample size formula for comparing Drug A to Drug B (in each group) is derived as

$$\begin{aligned} n_\mathrm{A} =n_\mathrm{B} =\frac{2\sigma _\mathrm{c}^{2} (z_{\alpha /2} +z_{\beta })^2}{k\delta _\mathrm{c}^{2}}(1-\rho ), \end{aligned}$$

where *k* is the number of cross-over periods; $\sigma _\mathrm{c}$, $\delta _\mathrm{c}$ are standard errors and treatment differences, respectively, and $\rho$ is the within-subject correlation. As can be seen, the more repeats of the cross-over, the less sample size will be needed for detecting the difference between A and B. Also the higher the correlation is (assuming $r>0$), the less sample size will be needed.

For determining whether assigning patients to Drug A or Drug B, one can set a threshold according to clinical criteria, say 10 %. For example, if the overall efficacy score under A is 10 % better than B, then assign the subject to Drug A in extension; if the overall efficacy score under B is 10 % better than A, then assign the subject to Drug B in extension; if the overall efficacy scores between A and B are within 10 %, the subject can be randomly assigned to either A or B.

For the extension phase, the total sample size (combined active groups A and B) formula for comparing Drug A to Drug B is derived as

$$\begin{aligned} n_\mathrm{AB} =\frac{\sigma _\mathrm{e}^{2} (z_{\alpha /2} +z_{\beta })^{2}}{r(1-r)\delta _\mathrm{e}^{2}}, \end{aligned}$$

where $\sigma _\mathrm{e}$ and $\delta _\mathrm{e}$ are standard errors and treatment differences in extension phase, respectively; and *r* is the proportion of patients assigned to Drug A. If $r = 0.5$ (the same proportion to A or B), then the sample size formula is the same as the usual two-sample parallel design with 1:1 allocation ratio. From practical point of view, if $r>0.75$, i.e., more than 75 % of the patients were in favor of A, one can easily conclude that A is a favorable treatment (Drug) for majority of patients. In other words, you may not need the extension phase to draw the conclusion. Therefore, for planning purpose, we could restrict $0.5 \le r \le 0.75$ in the N-of-1 design.

Finally, we take the maximum of the two sample sizes to get the sample size for the entire study.

$$\begin{aligned} n_\mathrm{AB} =\max \left\{ \frac{4\sigma _\mathrm{c}^{2}}{k\delta _\mathrm{c}^{2}}(1-\rho ),\;\frac{\sigma _\mathrm{e}^{2}}{r(1-r)\delta _\mathrm{e}^{2}}\right\} {(z_{\alpha /2} +z_{\beta })^{2}}. \end{aligned}$$

If the study included a placebo arm, we need to consider the sample size for placebo group. If the allocation ratio of active sequences (A/B and B/A) to placebo sequence (P/P) is 1:1:1, we obtain the sample size for placebo:

$$\begin{aligned} n_\mathrm{P} = \left( \frac{1+2r}{2r}\right) \frac{\sigma _\mathrm{e}^{2} (z_{\alpha /2}+z_{\beta })^{2}}{\delta _\mathrm{e}^{2}}. \end{aligned}$$

With these formulas, one can plan the N-of-1 study according to the study objectives by assuming different study parameters (i.e., $k, r, \sigma _{\mathrm {c}}$, $\delta _{\mathrm {c,}}\,\sigma _{\mathrm {e}}$, and $\delta _{\mathrm {e}})$.

### Efficacy Analysis Consideration

The proportion (*r*) of patients distributed to Drug A at the end of cross-over phase could be an important qualitative variable for assessing whether these two drugs are (1) no difference or (2) one is superior to the other. We can form a hypothesis test with $H_{0}$: $r = 50$ % (i.e., A $=$ B) versus $H_{1}$: $r > 50~\% + p$ to test whether more patients will stay with Drug A compared to B. This could be the first step of analyzing the differences by simply applying the $\chi ^{2}$ test. However, this provides only the qualitative evaluation, not the quantitative evaluation.

The primary objective of the design is to identify the optimal treatment for a long-term therapy. The efficacy measurements (safety as well) are collected during the cross-over phase as well as the extension phase. We need to combine the data in both phases together to assess the overall effect. To do so, we need to derive a composite variable that is able to carry the information gathered in both phases.

### Composite Efficacy Measure

In cross-over phase, each patient undergoes two different treatments. Thus in extension phase, whether to continue on Drug A or B is a conditional random variable I. We could define an overall endpoint by combining the efficacy measurements from both cross-over and extension phases.

Let $X^\mathrm{A}$ and $X^\mathrm{B}$ denote the efficacy measurement at cross-over phase for Drug A and Drug B; *Y* denote the efficacy measurement at extension phase; $I^\mathrm{A}$, $I^\mathrm{B}$ denote the indicators whether the patient is assigned to Drug A or Drug B; Z denote the overall efficacy measurement. Thus, we got the following expression for *Z*:

$$\begin{aligned} Z^\mathrm{A} = X^\mathrm{A} + Y^*I^\mathrm{A}; \quad Z^\mathrm{B} = X^\mathrm{B}+Y^*I^\mathrm{B}, \\ \end{aligned}$$

where $I^\mathrm{A}$ is 1 if A is assigned and 0 otherwise; $I^\mathrm{B}$ is 1 if B is assigned and 0 otherwise.

### Test Statistics

- *Comparing Drug A to Drug B* Let $x_{ij}^\mathrm{A}$, $x_{ij}^\mathrm{B}$ denote the outcome of *i*th patient at *j*th cross under treatment A or B; $y_{i}^\mathrm{A}$, $y_{i}^\mathrm{B}$ denote the outcome during extension phase for *i*th patient under Drug A or Drug B. Then we have the overall score for *i*th patient as the following:
  $$\begin{aligned} Z_{i}^\mathrm{A} =\frac{1}{k}\sum _{j=1}^k {x_{ij}^\mathrm{A} +y_{i}^\mathrm{A}} I_{i}^\mathrm{A}\; \hbox {and}\; Z_{i}^\mathrm{B} =\frac{1}{k}\sum _{j=1}^k {x_{ij}^\mathrm{B} +y_{i}^\mathrm{B}} (1-I_{i}^\mathrm{A}), \end{aligned}$$
  where *k* is the number of cross-over. Thus we have the treatment difference between A and B:
  $$\begin{aligned} \Delta _\mathrm{AB}&=\frac{1}{n}\sum _{i=1}^n {Z_{i}^\mathrm{A}} -\frac{1}{n}\sum _{i=1}^n {Z_{i}^\mathrm{B}} =\frac{1}{n}\sum _{i=1}^n {\frac{1}{k}} \sum _{j=1}^k {\left( {x_{ij}^\mathrm{A} -x_{ij}^\mathrm{B}} \right) } \nonumber \\&\quad +\,\frac{1}{n}\sum _{i=1}^n {\left( {y_{i}^\mathrm{A} I_{i}^\mathrm{A} -y_{i}^\mathrm{B} (1-I_{i}^\mathrm{A})} \right) }\;\text {or}\\ \Delta _{AB}&= \underbrace{(\bar{{X}^A}-\bar{{X}^B})}_{(1)}+\underbrace{(\bar{{Y}^A}I-\bar{{Y}^B}(1-I))}_{(2)}, \end{aligned}$$
  where $$\bar{{Y}^A}=\frac{1}{n}\sum \limits _{i=1}^n {y_{i}^\mathrm{A}}\; \hbox {and}\; \bar{{Y}^B}=\frac{1}{n}\sum \limits _{i=1}^n {y_{i}^\mathrm{B}}$$.
  Parts (1) and (2) are the differences contributed by cross-over phase and extension phase, respectively.
  The expectation of$\Delta _\mathrm{AB} =\delta _\mathrm{c} +(\mu _\mathrm{A} r-\mu _\mathrm{B}(1-r))$. Under assumption of constant within-subject correlation, we have
  $$\begin{aligned} \text {Var}(\Delta _\mathrm{AB}) = \frac{2\sigma _\mathrm{c}^{2}}{nk}(1-\rho )+\frac{\sigma _\mathrm{A}^{2}}{n}r + \frac{\sigma _\mathrm{B}^{2}}{n}(1-r)+\frac{1}{n}(\mu _\mathrm{A} +\mu _\mathrm{B})^{2} r (1-r). \end{aligned}$$
  Thus we can formulate the overall test statistics for comparing Drug A versus Drug B.
  $$\begin{aligned} z_\mathrm{AB} =\frac{\hat{{\delta }}_\mathrm{c} +(\hat{{\mu }}_\mathrm{A} r-\hat{{\mu }}_\mathrm{B} (1-r))}{\sqrt{\frac{2\sigma _\mathrm{c}^{2}}{nk}(1-\rho )+\frac{\sigma _\mathrm{A}^{2}}{n}r+\frac{\sigma _\mathrm{B}^{2}}{n}(1-r)+\frac{1}{n}(\hat{{\mu }}_\mathrm{A} +\hat{{\mu }}_\mathrm{B})^{2} r (1-r)}}, \end{aligned}$$
  where *n* is the number of patients; *k* is the number of cross-over; $\hat{{\sigma }}_\mathrm{c}$, $\hat{{\sigma }}_\mathrm{A}$, $\hat{{\sigma }}_\mathrm{B}$ are sample standard errors; $\hat{{\delta }}_\mathrm{c}$ is the treatment difference between A and B in cross-over phase; $\hat{{\mu }}_\mathrm{A}$ and $\hat{{\mu }}_\mathrm{B}$ are the average effects of groups A and B in extension phase; $\rho$ is the correlation; *r* is the proportion of patients assigned to Drug A. The detailed derivations of formulas are given in Online Appendix.
- *Comparing active drug to placebo* We can write the sample means of Drugs A, B, and P as the following
  $$\begin{aligned} \bar{{Z}^{A}}&= \underbrace{\frac{1}{n}\sum _{i=1}^n {\frac{1}{k}} \sum _{j=1}^k {x_{ij}^\mathrm{A}}}_{(1)}+\underbrace{\frac{1}{n}\sum _{i=1}^n {y_{i}^\mathrm{A} I_{i}^\mathrm{A}}}_{(2)};\quad \bar{{Z}_{B}} =\underbrace{\frac{1}{n}\sum _{i=1}^n {\frac{1}{k}} \sum _{j=1}^k {x_{ij}^\mathrm{B}}}_{(1)}+\underbrace{\frac{1}{n}\sum _{i=1}^n {y_{i}^\mathrm{B} I_{i}^\mathrm{B}}}_{(2)}; \nonumber \\ \bar{{Z}^{P}}&=\underbrace{\frac{1}{m}\sum _{i=1}^m {\frac{1}{2k}} \sum _{j=1}^{2k} {x_{ij}^\mathrm{P}}}_{(3)}+\underbrace{\frac{1}{m}\sum _{i=1}^m {y_{i}^\mathrm{P}} }_{(4)}. \end{aligned}$$
  We got
  $$\begin{aligned} E(\Delta _\mathrm{AP})=E(\bar{{X}^{A}}-\bar{{X}^{P}})+E(\bar{{Y}^{A}}I-\bar{{Y}^{P}})=\hat{{\delta }}_\mathrm{c} +\hat{{\mu }}_\mathrm{A} r-\hat{{\mu }}_\mathrm{P} \end{aligned}$$
  $$\begin{aligned} \text {Var}(\bar{{Z}^{A}})&= \text {Var}((1))+\text {Var}((2))+2^*\text {cov}((1),(2)) \\&=\frac{1}{nk}\sigma ^2 (1+(k-1)\rho )+\frac{1}{n}(\sigma ^{2} r+\mu _\mathrm{A}^{2} r(1-r))+\frac{2}{n}\sigma ^{2} \rho r; \end{aligned}$$
  and
  $$\begin{aligned} \text {Var}(\bar{{Z}^{P}})=\frac{1}{2mk}\sigma ^{2} (1+(2k-1)\rho )+\frac{1}{m}\sigma ^{2} (1+2\rho ). \end{aligned}$$
  Finally, we got the test statistics for comparing Drug A to placebo as
  $$\begin{aligned} z_\mathrm{AP} =\frac{\hat{{\delta }}_\mathrm{c} +\hat{{\mu }}_\mathrm{A} r-\hat{{\mu }}_\mathrm{P}}{\sqrt{\frac{1}{nk}\sigma ^2(1+(k-1)\rho )+\frac{1}{n}(\sigma ^2 r+\mu _\mathrm{A}^{2} r(1-r))+\frac{2}{n}\sigma ^2 \rho r+\frac{1}{2mk}\sigma ^2(1+(2k-1)\rho ) + \frac{1}{m}\sigma ^2 (1+2\rho )}}. \end{aligned}$$
  With the same approach, we can get the test statistics for comparing Drug B to placebo.

**Table 1:** Patient population and rate of responses to study drugs

Study population (*W*)	Drug A	Drug B	Placebo (P)
Sub-population *U*	0.5	0.1	0.1
Sub-population *V*	0.1	0.5	0.1

**Table 2:** Power increase in simulation

*r* (proportion to A)	$\rho$ (correlation)	*k* (no. of cross-overs)	Power (A vs. P)	Power (B vs. P)
0.5	0.3	1	0.906	0.905
0.5	0.3	2	0.957	0.949
0.5	0.3	3	0.964	0.971
0.5	0.3	4	0.984	0.981

**Table 3:** Type I error inflation in simulation

*r* (proportion to A)	$\rho$ (correlation)	*k* (no. of cross-overs)	Type I error
0.5	0.3	1	0.090
0.5	0.3	2	0.092
0.5	0.3	3	0.097
0.5	0.3	4	0.098
0.5	0.8	1	0.073
0.5	0.8	2	0.070
0.5	0.8	3	0.072
0.5	0.8	4	0.075

### Simulation

A simulation study was performed using R (see R scripts in Online Resource). As previously stated, the entire study population contains two subgroups U and V, which react differently on drugs A and B. We assumed that for population U, Drug A has full effect 0.5 but Drug B only has effect 0.1 (Table 1). For population V, the situation is the opposite. And placebo effects on two populations are both 0.1.

We calculated the sample size for traditional parallel design of comparing Drug A versus placebo or Drug B versus placebo, which is $n=98$ per group, to obtain power $=$ 80 %. Using this sample size, we calculated the power using our new design by performing simulation 1000 times. Apparent power increase has been observed (Table 2). And slight type I error inflation was observed, compared with 0.05 (Table 3). Most likely, the source of inflation may come from the over-selection for subjects’ assignment based on better scores in the cross-over phase.

Some additional simulation results with various different parameters were also summarized in Tables 4 and 5. From Table 4, we could see that the power decreases as correlation increases.

**Table 4:** Simulation results with various $\rho$ (correlation)

*r* (proportion to A)	$\rho$ (correlation)	*k* (no. of cross-overs)	Power (A vs. P)	Power (B vs. P)
0.5	0	3	0.997	0.999
0.5	0.1	3	0.998	0.996
0.5	0.2	3	0.990	0.988
0.5	0.3	3	0.977	0.976
0.5	0.4	3	0.966	0.957
0.5	0.5	3	0.953	0.949
0.5	0.6	3	0.918	0.913
0.5	0.7	3	0.923	0.912
0.5	0.8	3	0.917	0.910
0.5	0.9	3	0.871	0.874
0.5	1.0	3	0.867	0.862

**Table 5:** Simulation results with various *r* (proportion to A)

*r* (proportion to A)	$\rho$ (correlation)	*k* (no. of cross-overs)	Power (A vs. P)	Power (B vs. P)	*N* (sample size)
0.2	0.3	3	0.684	1.00	613
0.3	0.3	3	0.879	0.903	273
0.4	0.3	3	0.879	0.964	154
0.5	0.3	3	0.968	0.977	98
0.6	0.3	3	0.988	0.703	69
0.7	0.3	3	0.977	0.255	50
0.8	0.3	3	0.983	0.069	39

In Table 5, we simulated the cases when *r* (proportion to A) varies, which are closer to the conditions in the real world, where the proportion to one subgroup cannot be always 0.5. The sample sizes used here were calculated based on traditional parallel design of comparing Drug A versus placebo to obtain power $=$ 80 %. As suggested in this table, increasing of *r* leads to the enhancement of power of A versus placebo, and the gain of power (compared to traditional design) starts from $r = 0.3$.

## A Case Report

One of the important utilizations of our new design is the clinical trials with traditional Chinese medicine (TCM). TCM has been used for thousands of years and plays an important role in treating various diseases in China and Asian countries. However, it is still not widely accepted due to lack of solid evidence to support its efficacy claims [35]. Doctors may prescribe TCM treatment by adding or removing certain components according to observation of individual patient’s characteristics, symptoms, pulse, color of tong, etc. This highly complies with the idea of personalized medication/treatment, but it also leads to some challenges in conducting a traditional randomized clinical trial. Thus the N-of-1 design, which is a combination of personalized clinical trial and randomized clinical trial, could play a critical role here. Recently there are several ongoing TCM clinical trials using the N-of-1 design. We have been involved in a Chinese National 11th 5-Year Plan Research Project to evaluate personalized TCM treatment to patients with diabetic retinopathy and glaucoma. This study used the N-of-1 design to identify the best treatment to the patients and in an extension phase let patients continue on the best treatment identified.

### Background

The objective of the study is to use our new design to study methodology in design and evaluation of TCM clinical trials. The study population includes the patients aging 35–75 with diabetic retinopathy and glaucoma. We selected two TCM drugs: one with effect in treating this disease (treatment A) and one with no effect in treating this disease (treatment B, placebo).

### Treatment Procedure

This study involved Drug A (the drug of interest) and Drug B (the placebo). There were two cross-over periods ($k=2$). In each cross-over period, a patient took treatments in sequence A/B or B/A. The sequence of treatments within a period for each patient was randomly generated using SAS procedure “proc plan.”

There were 10 weeks in each cross-over period. In the first 4 weeks, patients took the first drug and the 5th week was the wash-out period; in weeks 6th–9th, patients took the second drug and the 10th week was the wash-out period. In the 5th and 10th week, treatment efficacy information was collected. After evaluating the cross-over period, a better drug (Drug A or Drug B) was selected for each patient and the patient took this drug for 12 weeks.

### Efficacy Endpoints

Short-term efficacy measurements included physician’s symptom assessments and patient self-symptom questionnaires. Long-term efficacy measurements included vision, fundus fluorescein angiography, FERG and OPs, TCM symptom scales, and VFQ-25 scales.

An important criterion is ART (Ratio of Drug A), which is the ratio of patients staying in group A in extension phase. If $\text {ART} \ge 70$ %, we could identify that Drug A has significant effects; if 50 % < ART < 70 %, Drug A has some effects; if $\text {ART} \le 50$ %, Drug A has no effects at all.

The drug efficacy is measured by scores determined by physician and patient, with the lower score, the better symptom.

### Results

One problem of this study is the missing data. We planned to recruit 60 patients, but only 41 patients participated at the beginning of cross-over phase. After cross-over phase was complete, only 35 patients remained in the study with 20 of them assigned to Drug A and 15 of them to Drug B (ART$=$57.1 %). And only 28 patients completed both cross-over phase and extension phase, with 16 in group A and 12 in group B (r$=$57.1 %). According to ART which is between 50 and 70 %, Drug A has some effects.

The correlation at cross-over phase was $\rho =0.69$. The mean score at cross-over phase for Drug A is $-10.0$; the mean score at cross-over phase for Drug B is $-9.0$; the mean score at extension phase for Drug A is $-2.4$; the mean score at extension phase for Drug B is $-0.7$. The standard error at cross-over phase is 12.94; the standard error at extension phase for Drug A is 10.45; the standard error at extension phase for Drug B is 10.10. Calculated test statistics z is $-0.9605$, leading to a two-sided *p* value = 0.337. This *p* value indicates that Drug A is not significantly better than Drug B (placebo).

## Discussion

The new N-of-1 design described in this manuscript provides a method to study the personalized treatment. The nature of N-of-1 design determines that the patient serves as his/her own control, making the results more reliable. It is a design that the treatment is not determined by somebody else like the traditional “play-the-winner” approach, where *i*th subject’s treatment was determined by the success of the treatment on ($i-1$)th subject [36, 37]. It is also different from the “patients-like-me” approach, in which the k most similar neighbors are examined sequentially until a statistically significant conclusion can be drawn [38]. Comparing to personalized treatment approaches based on genomic profile or biomarkers, the design is much simpler and easier to conduct. The design is especially suitable for trials of chronic diseases where patient’s baseline characteristic is hard to be determined or where the treatment effect may vary among patients, such as in TCM.

There are a few more advantages of this new design. First of all, this design is beneficial to the patients: in relatively early cross-over phase, we could easily identify which treatment actually works better for a particular patient, so in the extension phase the patient could be treated with a treatment which was proved to work better for him/her. This design also maximizes relative treatment effects by correctly assigning patients to corresponding drugs, thus reducing the chance that an effective treatment (which might be effective to only subgroups of patients) being abandoned simply due to lack of significance across the total population (which might be due to the dilution by certain unknown prognostic factors). Thus this design could not only find best treatments for individual patients but also evaluate general treatment effects for larger subgroups. And the advantage of power increase indicates the potential of recruiting fewer patients, thus cutting the cost and saving the time. Finally, the patient information gathered within subgroups might also provide a chance for the researchers to further summarize the common characteristics of subgroup patients, which might make it possible to gain better understanding toward the detailed working mechanism of a certain treatment.

There are also some limitations of this design. The disadvantage is that the additional cross-over phase will prolong the duration of study. There is slight Type I error inflation. According to the results shown in Table 3, increasing the number of cross-over could partially suppress the inflation. The carry-over effects in cross-over phase need to be controlled well, for example, by increasing wash-out time, to avoid confounding the trial results.

## Electronic supplementary material

Below is the link to the electronic supplementary material.

Supplementary material 1 (docx 89 KB)
