# Supplementary material for: N-of-1 Design and Its Applications to Personalized Treatment Studies
Source: Stat Biosci. 2016 Sep 6;9(2):662–75. doi: 10.1007/s12561-016-9165-9 (PMC5711967; doi:10.1007/s12561-016-9165-9)
Supplement: Supplementary file 1 — Supplementary material 1 (docx 89 KB) [file 12561_2016_9165_MOESM1_ESM.docx]

Article title: N-of-1 Design and its Applications to Personalized Treatment Studies

Journal Name: Statistics in Biosciences

Author names: Tailiang Xie and Zhuoxin Yu

Affiliation: Brightech International, 285 Davidson Avenue, Somerset, NJ 08873

Corresponding Author: Tailiang Xie [tomx@brightech-intl.com](mailto:tomx@brightech-intl.com)

This online resource contains R scripts for simulation and the formula derivations.

There are two R scripts included here: one is the function script; the other one is the main script. All the R functions in the function script need to be run before running the main script.

There are three sessions in formula derivations: Sample Size, Difference of Effects between Drug A and Drug B, and Difference of Effects between Drug A and Placebo.

**R Function Script:**

##Load Package "MASS"

library("MASS")

##Traditional Randomization

RandTrad<-function(EffTrad){

n=dim(EffTrad)[1]

RdNum<-runif(n)

EffRd<-cbind(EffTrad,RdNum)[order(RdNum),]

TrtA<-EffRd[c(1:(n/3)),1]

TrtB<-EffRd[c((n/3+1):(n*2/3)),2]

TrtPl<-EffRd[c((n*2/3+1):n),3]

TrtTrad<-data.frame(cbind(TrtA,TrtB,TrtPl))

return(TrtTrad)

}

##Generate Sample Efficacy Values for Traditional Randomization

EfficacyTrad<-function(n,p,muPl,muXA,muXB,muYA,muYB,sigma){

Pl<-rnorm(n,muPl,sigma)

XA<-rnorm(round(n*p),muXA,sigma)

XB<-rnorm(round(n*p),muXB,sigma)

YA<-rnorm(n-round(n*p),muYA,sigma)

YB<-rnorm(n-round(n*p),muYB,sigma)

#Efficacy Matrix

EffTrad<-data.frame(cbind(c(XA,YA),c(XB,YB),Pl))

names(EffTrad)<-c("Drug_A","Drug_B","Placebo")

return(EffTrad)

}

##Generate Sample Efficacy Values for Cross-over, with Correlation

Efficacy<-function(n,p,muPl,muXA,muXB,muYA,muYB,sigma,cr,rho){

m=(cr+1)*2

SigmaMatrix <- matrix(c(rep(c(sigma^2,rep(sigma^2*rho,m)),m-1),sigma^2),m,m)

MVR_X<-mvrnorm(round(n*p), rep(c(muXA, muXB),m/2), SigmaMatrix)

MVR_Y<-mvrnorm(n-round(n*p), rep(c(muYA, muYB),m/2), SigmaMatrix)

MVR_Pl<-mvrnorm(n, rep(muPl,m), SigmaMatrix)

XA<-data.frame(matrix(data=NA,nrow=round(n*p),ncol=cr+1))

XB<-data.frame(matrix(data=NA,nrow=round(n*p),ncol=cr+1))

YA<-data.frame(matrix(data=NA,nrow=n-round(n*p),ncol=cr+1))

YB<-data.frame(matrix(data=NA,nrow=n-round(n*p),ncol=cr+1))

Pl=data.frame(MVR_Pl)

for(i in 1:cr){

XA[,i]<-MVR_X[,i*2-1]

XB[,i]<-MVR_X[,i*2]

YA[,i]<-MVR_Y[,i*2-1]

YB[,i]<-MVR_Y[,i*2]

names(XA)[i]<-paste("Cros_A",i)

names(XB)[i]<-paste("Cros_B",i)

names(YA)[i]<-paste("Cros_A",i)

names(YB)[i]<-paste("Cros_B",i)

names(Pl)[i]<-paste("Cros_Pl",i)

names(Pl)[cr+i]<-paste("Cros_Pl",cr+i)

}

XA[,cr+1]<-MVR_X[,cr*2+1]

XB[,cr+1]<-MVR_X[,cr*2+2]

YA[,cr+1]<-MVR_Y[,cr*2+1]

YB[,cr+1]<-MVR_Y[,cr*2+2]

names(XA)[cr+1]<-"Ext_A"

names(XB)[cr+1]<-"Ext_B"

names(YA)[cr+1]<-"Ext_A"

names(YB)[cr+1]<-"Ext_B"

names(Pl)[cr*2+1]<-"Ext_Pl"

Eff<-data.frame(cbind(rbind(XA,YA),rbind(XB,YB),Pl))

#True Flag of Patients

Eff$FlagT<-"NULL"

Eff$FlagT[c(1:round(n*p))]<-"A"

Eff$FlagT[c((round(n*p)+1):n)]<-"B"

return(Eff)

}

##Assignment after Cross-over if cr>1

RandCros<-function (Eff) {

n=dim(Eff)[1]

RdNum<-runif(n)

EffRd<-cbind(Eff,RdNum)[order(RdNum),]

#Calculate Means during Cross-over

EffRd$MeanCrosA=rowMeans(EffRd[,c(1:cr)])

EffRd$MeanCrosB=rowMeans(EffRd[,c((cr+2):(cr*2+1))])

#Assignment Flag

for (i in 1:dim(EffRd)[1])

if (EffRd$MeanCrosA[i]>=EffRd$MeanCrosB[i]){

EffRd$FlagA[i]<-"A"

}else{

EffRd$FlagA[i]<-"B"

}

#Placebo

TrtPl<-data.frame(EffRd[c((round(n/2)+1):n),c(((cr+1)*2+1):(cr*4+3))])

#Drug A and Drug B

EffRdAB<-EffRd

TrtA<-EffRdAB[EffRdAB$FlagA=="A",c(1:((cr+1)*2),(cr+1)*4+1,c(((cr+1)*4+3):((cr+1)*4+5)))]

TrtB<-EffRdAB[EffRdAB$FlagA=="B",c(1:((cr+1)*2),(cr+1)*4+1,c(((cr+1)*4+3):((cr+1)*4+5)))]

Trt<-list(TrtA, TrtB, TrtPl)

names(Trt)<-c("TrtA","TrtB","TrtPl")

return(Trt)

}

##Assignment after Cross-over if cr=1

RandCros_cr1<-function (Eff) {

n=dim(Eff)[1]

RdNum<-runif(n)

EffRd<-cbind(Eff,RdNum)[order(RdNum),]

#Calculate Means during Cross-over

EffRd$MeanCrosA=EffRd[,1]

EffRd$MeanCrosB=EffRd[,3]

#Assignment Flag

for (i in 1:dim(EffRd)[1])

if (EffRd$MeanCrosA[i]>=EffRd$MeanCrosB[i]){

EffRd$FlagA[i]<-"A"

}else{

EffRd$FlagA[i]<-"B"

}

#Placebo

TrtPl<-data.frame(EffRd[c((round(n/2)+1):n),c(((cr+1)*2+1):(cr*4+3))])

#Drug A and Drug B

EffRdAB<-EffRd

TrtA<-EffRdAB[EffRdAB$FlagA=="A",c(1:((cr+1)*2),(cr+1)*4+1,c(((cr+1)*4+3):((cr+1)*4+5)))]

TrtB<-EffRdAB[EffRdAB$FlagA=="B",c(1:((cr+1)*2),(cr+1)*4+1,c(((cr+1)*4+3):((cr+1)*4+5)))]

Trt<-list(TrtA, TrtB, TrtPl)

names(Trt)<-c("TrtA","TrtB","TrtPl")

return(Trt)

}

**R Main Script:**

#First of all load all the R functions in R function script

#Three groups: Drug A, Drug B and Placebo.

#Efficacy of Placebo = 0.1

#First population X: Response to Drug A = 0.5 and Response to Drug B = 0.1

#Second population Y: Response to Drug A = 0.1 and Response to Drug B = 0.5

muXA<-0.5

muXB<-0.1

muYA<-0.1

muYB<-0.5

muPl<-0.1

sigma<-0.5

za<-1.96 #Type I error=0.05

zb<-0.84 #Power=0.8

p<-0.5 #Proportion of patients for population X, could be varied

muA<-p*muXA+(1-p)*muXB

muB<-p*muXB+(1-p)*muYB

nE<-ceiling(2*sigma^2*(za+zb)^2/(muA-muPl)^2)

n<-nE*3

cr<-4 #Number of cross-over, could be varied

rho<-0.3 #Correlation, could be varied

#For Type I Error only

muXA<-0.1

muXB<-0.1

muYA<-0.1

muYB<-0.1

muA<-0.1

muB<-0.1

#Tradiational Randomization

#Test power

asim<-1000

pv<-matrix(data=NA,nrow=asim,ncol=3)

for(i in 1:asim)

{

EffTrad=EfficacyTrad(nE,p,muPl,muXA,muXB,muYA,muYB,sigma)

#TrtTrad<-RandTrad(EffTrad)

muA_Trad<-mean(EffTrad$Drug_A)

muB_Trad<-mean(EffTrad$Drug_B)

muPl_Trad<-mean(EffTrad$Placebo)

sdA_Trad<-sd(EffTrad$Drug_A)

sdB_Trad<-sd(EffTrad$Drug_B)

sdPl_Trad<-sd(EffTrad$Placebo)

Zap<-(muA_Trad-muPl_Trad)/((sigma^2/nE+sigma^2/nE)^0.5)

Zbp<-(muB_Trad-muPl_Trad)/((sigma^2/nE+sigma^2/nE)^0.5)

Zab<-(muA_Trad-muB_Trad)/((sigma^2/nE+sigma^2/nE)^0.5)

pv[i,1]<-2*pnorm(-abs(Zap))

pv[i,2]<-2*pnorm(-abs(Zbp))

pv[i,3]<-2*pnorm(-abs(Zab))

}

mean(pv[,1]<0.05)

mean(pv[,2]<0.05)

mean(pv[,3]<0.05)

##N-of-1 New Design

#Test power

asim<-1000

AssPb<-rep(0,asim)

pv<-matrix(data=NA,nrow=asim,ncol=3)

for(i in 1:asim)

{

Eff=Efficacy(round(n*2/3),p,muPl,muXA,muXB,muYA,muYB,sigma,cr,rho)

if (cr>1){

Trt=RandCros(Eff)

}

if (cr==1){

Trt=RandCros_cr1(Eff)

}

TrtA<-Trt$TrtA

TrtB<-Trt$TrtB

TrtPl<-Trt$TrtPl

AssPb[i]<-(sum(TrtA$FlagT==TrtA$FlagA)+sum(TrtB$FlagT==TrtB$FlagA))/(dim(TrtA)[1]+dim(TrtB)[1])

MeanA_Cros<-mean(c(TrtA$MeanCrosA,TrtB$MeanCrosA))

MeanB_Cros<-mean(c(TrtA$MeanCrosB,TrtB$MeanCrosB))

MeanPl_Cros<-mean(rowMeans(TrtPl[,c(1:(cr*2))]))

n_Cros<-dim(TrtA)[1]+dim(TrtB)[1]

MeanA_Ext<-sum(TrtA$Ext_A)/n_Cros

MeanB_Ext<-sum(TrtB$Ext_B)/n_Cros

MeanPl_Ext<-mean(TrtPl$Ext_Pl)

m_Cros<-dim(TrtPl)[1]

#Calculate Z-value:

Eap<-MeanA_Cros-MeanPl_Cros+MeanA_Ext-MeanPl_Ext

Ebp<-MeanB_Cros-MeanPl_Cros+MeanB_Ext-MeanPl_Ext

Eab<-MeanA_Cros-MeanB_Cros+MeanA_Ext-MeanB_Ext

Vap<-sigma^2*(1+(cr-1)*rho)/(n_Cros*cr)+sigma^2*p*(1+2*rho)/n_Cros+muA^2*p*(1-p)/n_Cros+sigma^2*(1+(2*cr-1)*rho)/(2*m_Cros*cr)+sigma^2*(1+2*rho)/m_Cros #Updated

Vbp<-sigma^2*(1+(cr-1)*rho)/(n_Cros*cr)+sigma^2*p*(1+2*rho)/n_Cros+muB^2*p*(1-p)/n_Cros+sigma^2*(1+(2*cr-1)*rho)/(2*m_Cros*cr)+sigma^2*(1+2*rho)/m_Cros #Updated

Vab<-2*sigma^2*(1-rho)/(n_Cros*cr)+sigma^2/n_Cros+(muA+muB)^2*p*(1-p)/n_Cros

Zap<-Eap/Vap^0.5

Zbp<-Ebp/Vbp^0.5

Zab<-Eab/Vab^0.5

pv[i,1]<-2*pnorm(-abs(Zap))

pv[i,2]<-2*pnorm(-abs(Zbp))

pv[i,3]<-2*pnorm(-abs(Zab))

}

mean(AssPb)

mean(pv[,1]<0.05)

mean(pv[,2]<0.05)

mean(pv[,3]<0.05)

**Formula derivations:**

**Sample Size:**

In cross-over phase,

Let $x_{ij}^{A}$ be outcome of i^th^ patient, at j^th^ cross-over under treatment A,

Let be outcome of i^th^ patient, at j^th^ cross-over under treatment B,

$\delta_{ij}$ = treatment difference for i^th^ pt, at j^th^ cross-over.

Assume k denote number of cross-over for each patient,

$$Var\left(\delta_{ij}\right)=Var\left(x_{ij}^{A}-x_{ij}^{B}\right)=\sigma_{A}^{2}+\sigma_{B}^{2}-2*\mathrm{cov}\left(x_{ij}^{A},x_{ij}^{B}\right)=\sigma_{A}^{2}+\sigma_{B}^{2}-2\sigma_{A}\sigma_{B}\rho$$

where $\rho=\frac{\mathrm{cov}\left(x_{ij}^{A},x_{ij}^{B}\right)}{\sigma_{A}\sigma_{B}}$ is correlation between A and B.

The mean difference over all subjects is estimated by

$$\hat{\delta}_{c}=\frac{1}{n}\sum_{i=1}^{n} \frac{1}{k}\sum_{j=1}^{k} \delta_{ij}$$

Also assuming no correlation between different cross-overs (since we assume enough “wash out period” ) thus

Further assume == (variance at cross-over phase) then

We get the sample size formula

$$n_{A}=n_{B}=\frac{2\sigma_{c}^{2}(z_{\alpha/2}+z_{\beta})^{2}}{k\delta_{c}^{2}}(1-\rho)$$

In extension phase, assume (total patients at cross-over phase).

$$\sigma_{\delta}^{2}=\frac{\sigma_{A}^{2}}{n_{A}}+\frac{\sigma_{B}^{2}}{n_{B}}$$

Assume ==

Where $r=\frac{n_{A}}{n_{AB}}$ (proportion of patients to A)

(proportion of patients to B)

So

If (A/B):(B/A):(P/P) = 1:1:1

In extension phase,

Assume ==

$$n_{P}=(\frac{1+2r}{2r})\frac{\sigma_{e}^{2}(z_{\alpha/2}+z_{\beta})^{2}}{\delta_{e}^{2}}$$

**Difference of Effects between Drug A and Drug B**

Define for overall score:

where and are average score of subject i during cross-over period under treatment A, B respectively.

is the score of i^th^ patient in extension period under A. is an indicator of whether A is assigned to subject i. You can assume that

Let and

It can be shown that, for (1)

For (2), there is a formula that

Here

where and

Since and

In which,

So for

So For (2)

We also need to find out cov( (1), (2) )

For (a)

If we assume (is constant)

Similarly, we’ll have that

Thus cov((1),(2))=0

Therefore

So the test statistics is

**Difference of Effects between Drug A and Placebo**

We know that

Here

So

So

So
